# The effect of two lottery-style incentives on response rates to postal questionnaires in a prospective cohort study in preschool children at high risk of asthma: a randomized trial

**DOI:** 10.1186/1471-2288-12-186

**Published:** 2012-12-18

**Authors:** Lonneke B van der Mark, Karina E van Wonderen, Jacob Mohrs, Patrick JE Bindels, Milo A Puhan, Gerben ter Riet

**Affiliations:** 1 Department of General Practice, Academic Medical Center, Amsterdam, the Netherlands; 2 Department of General Practice, Erasmus Medical Center, Rotterdam, the Netherlands; 3 Department of Epidemiology, Johns Hopkins Bloomberg School of Public Health, Baltimore, USA

**Keywords:** Incentive, Longitudinal cohort study, Loss to follow up, Postal questionnaire, Randomized controlled trial, Response rate

## Abstract

**Background:**

In research with long-term follow-up and repeated measurements, quick and complete response to questionnaires helps ensure a study’s validity, precision and efficiency. Evidence on the effect of non-monetary incentives on response rates in observational longitudinal research is scarce.

**Objectives:**

To study the impact of two strategies to enhance completeness and efficiency in observational cohort studies with follow-up durations of around 2 years.

**Method and intervention:**

In a factorial design, 771 children between 2 and 5 years old and their parents participating in a prospective cohort study were randomized to three intervention groups and a control group. Three types of lotteries were run: (i) daytrip tickets for the whole family to a popular amusement park if they returned all postal questionnaires, (ii) €12.50-worth gift vouchers for sending back the questionnaire on time after each questionnaire round and (iii) a combination of (i) and (ii).

**Main outcome measures:**

Primary outcome was the proportion of participants who returned all questionnaires without any reminder. Secondary outcomes were ‘100% returned with or without reminder’, ‘probability of 100% non-response’, ‘probability of withdrawal’, ‘proportion of returned questionnaires’ and ‘overall number of reminders sent’.

**Statistical analysis:**

After testing for interaction between the two lottery interventions, the two trials were analysed separately. We calculated risk differences (RD) and numbers needed to “treat” and their 95% confidence intervals.

**Results:**

Daytrip nor voucher intervention had an effect on the proportion of participants who returned all questionnaires (RD −0.01; 95% CI-0.07 – 0.06) and (RD 0.02; 95% CI-0.50 – 0.08), respectively. No effects were found on the secondary outcomes.

**Conclusion:**

Our findings do not support the idea that lottery-style incentives lead to more complete response to postal questionnaires in observational cohort studies with repeated data collection and follow-up durations of around 2 years.

## Background

In longitudinal research, participant retention is of key importance but may be challenging. In particular, individuals who participate in observational research often do not directly benefit whilst their investment of time and effort may be considerable. Maintaining contact with participants for the time of the study to ensure valid results requires dedication and endurance from study personnel. What is the role of incentives to keep participants on board?

Booker et al. reviewed 11 randomized trials (RCT) on retention strategies in prospective population-based cohort studies with health outcomes [1]. One of the four RCTs evaluated the effect of a monetary (cash or check), one the effect of a non-monetary incentive (pencil) on retention rates of postal questionnaires [2,3]. Booker et al. concluded that it is unclear whether cash incentives are more effective than gifts of similar value. Doody et al. conducted a RCT in an adult cohort (n = 146,000) to estimate cancer risk among radiologic technicians [2]. Follow-up questionnaires were sent after 8 to 10 years and the monetary incentives were a USD 1 bill, a USD 2 bill or a check worth USD 5. Response rates were significantly higher in all incentive groups (24.6%, 28.9% and 27.5%, respectively) compared to no incentive (16.6%). White et al. included a pencil in a follow-up mailing sent 2 years after men and women completed a baseline cohort study questionnaire [3]. The response rate for the intervention group was 44% and 24% for the control group (p = 0.02). We were especially interested in the effects of somewhat larger and seemingly attractive incentives in which a lottery determined who actually won.

The trial we present here was nested in the ARCADE prospective cohort study in young children at high risk of developing asthma. Its primary objective is the construction of a clinical prediction rule for the diagnosis of asthma in young children in primary care. At baseline of ARCADE we randomized the 771 children to one of three lottery-style strategies (or control) aimed at increasing response, logistical efficiency and retention [4].

## Methods

### Participants and setting

All children admitted to the ARCADE cohort were eligible for participating in the trial, which was carried out as a substudy of ARCADE. ARCADE participants were recruited from 14 general practices in 2004, in The Netherlands. Admission criteria and baseline characteristics have been published elsewhere [4]. Briefly, children were between 1 and 5 years old had a high risk of developing asthma. Children were identified by an electronic search in computerized records of the general practitioners. 771 children’s parents signed the informed consent and participated in ARCADE. They were followed until the age of 6 years.

### Interventions and control

There were three intervention arms and one no-intervention control arm. In the first intervention group (V), ten €12.50-worth gift vouchers were raffled after each questionnaire round among participants who returned a completed questionnaire within 2 weeks of the date of sending. Ten participants in the second intervention group (VD) had a chance to receive a €12.50-worth gift voucher and, additionally, four families could receive a daytrip to the popular Dutch amusement park ‘Efteling’ with the whole family, if they had returned all questionnaires at the end of the study irrespective of the number of reminders. The third intervention group (D) could only receive the above-mentioned daytrip, but no gift voucher. The fourth group (C) was the no incentives control group.

In the daytrip trial, groups D and VD together were classified as intervention group ‘offered a daytrip’, and groups V and C together served as a reference group not offered a daytrip. Similarly, in the gift voucher trial, groups V and VD together were the intervention group ‘offered a gift voucher’ and groups D and C served as a reference group not offered a gift voucher.

Participants were randomized to one of the four groups at baseline for the duration of the follow up. The letters accompanying all questionnaires were for the most part identical for the four groups; except that the letters in the three intervention groups featured an additional paragraph, with a bold printed heading (see additional file for the letter (translated from Dutch into English).

The follow-up of the trial nested in the ARCADE study would last for 2 years, and participants received a maximum of 4 questionnaires, depending of their age at onset. For example, children older than 5 years at onset received only 2 questionnaires until they reached the age of 6 years.

Participants were informed at the start of the study about receiving a questionnaire every 6 months (T0, T6, T12 and T18). Every 12 months (T0 and T12) they received a questionnaire containing 130 multiple-choice questions about quality of life and airway problems [4]. In between (T6 and T18) they received a shorter questionnaire, containing 38 multiple-choice questions about the child’s health-related quality of life only. If a participant did not answer within 2 weeks, they received a postal reminder. Participants received a postal personalized letter and questionnaire consisting of a bright coloured cover and a stamped return envelope. The personalized letter had a logo of the academic hospital and the specific ARCADE study logo, both in red ink and signed by the researcher.

### Study design and randomization

**Figure 1:**
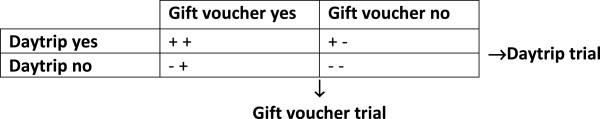
Study design.

The trial had a factorial design (see Figure 1). The four different groups were divided in 2 × 2 groups: Daytrip yes/no (daytrip trial) and gift voucher yes/no (gift voucher trial). An independent member of our staff (JM) assigned participants to one of the four groups according to a computer generated randomization list, with VisualBasic for Application in Microsoft Access. Randomization, stratified by general practice, was performed before sending the first questionnaire. We used a computer random number generator (the seed was the system’s timer) to select 4 random permuted blocks. The block size varied dependent on the number of patients with informed consent per practice location.

Patients were unaware of the trial and the group sizes, but were fully informed about ARCADE. The study was approved by the Dutch Central Committee on Research Involving Human Subjects (CCMO).

### Endpoints

The primary endpoint was the effect on overall response rate. For secondary outcomes we analyzed ‘proportion with 100% returned with or without reminder’, ‘probability of non-response’, ‘probability of withdrawal’, ‘percentage of returned questionnaires’ and ‘overall number of reminders sent’.

An additional analysis in the gift voucher trial was done on response rate per questionnaire. Outcome was calculated with repeated measurements logistic regression every 6 months.

All data were collected anonymously in an Access database. The dates of sending the questionnaire or reminder and receiving the questionnaire or reminder were noted.

### Statistical analysis

In total 15% of the values in the dataset (ranging from 6-42%) on covariates (level of education, allergy or asthma of the parents and IgE-serum results) were missing. The missing values were imputed using iterative chained equations (ICE; 20 imputation sets) [5]. These imputed data sets were used to be able to study potential intervention by intervention and intervention by covariate interactions with more power. Using the outcome ‘100% returned’, intervention interactions were assessed where we used alpha = 0.05 to define intervention interaction. We intended to analyse the data as two separate trials provided that there was no positive interaction between the two interventions. No positive interaction was found. Next, within each trial we assessed intervention-‘level of education’ interaction, intervention-ethnicity interaction, and interaction between intervention and the number of questionnaires (related to the child’s age at baseline) and all possible three-way interactions of those covariates to see, for example, if the intervention effects were different for respondents with low education and non-Dutch ethnicity. These interactions too were absent. We calculated risk differences and numbers needed to treat and their 95% confidence intervals without further adjustment for covariates. Proportions were tested using Chi-squared statistics (outcomes ‘100% returned’, 100% returned, no reminder’, ‘non-response’ and ‘withdrawal’), quantile regression of the median for the non-normally distributed variables (outcomes ‘number of reminders’ and ‘% returned’) and multilevel linear regression to take into account the repeated questionnaires every six months within each respondent.

All statistical analyses were performed with Stata 10.1 (Stata Corp., College Station, TX, USA).

## Results

Results were analyzed in the original, non-imputed, dataset, because there were no missing values in the intervention and outcome variables and the trials were analysed unadjusted.

**Figure 2:**
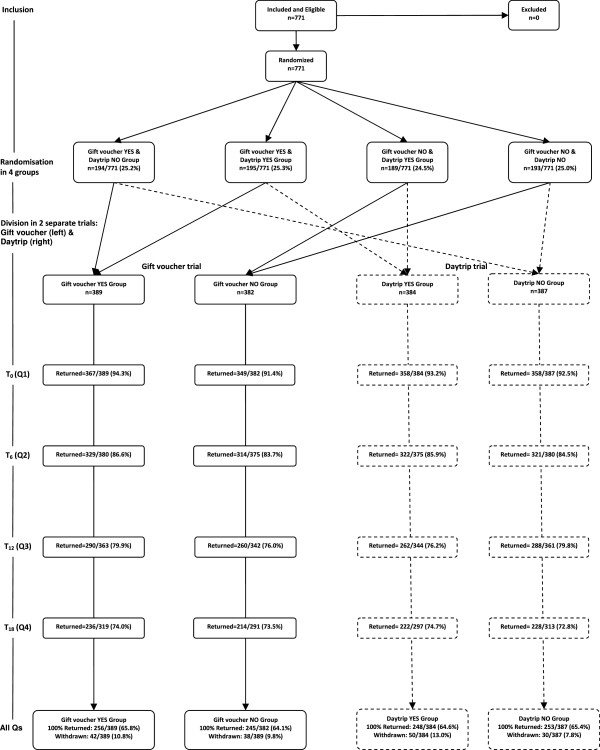
Flowchart of the Study.

Figure 2 shows the flowchart of all 771 children participating in the trial. Baseline comparisons on characteristics of the child participants and their parents, among the groups are presented in Tables 1 and 2. No differences were found among the groups on age, sex, severity of symptoms, family history, ethnicity and education level of the parents.

**Table 1:** Baseline characteristics of the daytrip trial

	**Daytrip**	**No Daytrip**	**Total**
**N**	384	387	771
**Male**, **n (%)**	207 (53.9)	225 (58.1)	432 (56)
**Age yrs**, **mean** (**SD**)*	2.25 (1.2)	2.29 (1.2)	2.27 (1.2)
**Nr of symptoms at onset**, **N (%)**			
**1** **	257 (66.9)	261 (67.4)	518 (67.2)
**2** ***	97 (25.3)	98 (25.3)	195 (25.3)
**3** ****	30 (7.8)	28 (7.2)	58 (7.5)
**Nr of Questionnaires**^#^			
**2**	21 (5.5)	17 (4.4)	38 (4.9)
**3**	42 (10.9)	42 (10.9)	84 (10.9)
**4**	321 (83.6)	328 (84.8)	649 (84.2)
**Level of education of parents**, **N (%)**			
**Lowest**	8 (2.1)	8 (2.1)	16 (2.1)
**Low**	56 (14.6)	61 (15.8)	117 (15.1)
**Medium**	144 (37.5)	146 (37.7)	290 (37.6)
**High**	149 (38.8)	148 (38.2)	297 (38.5)
**No information**	27 (7.0)	24 (6.2)	51 (6.6)
**Allergy or asthma parents**			
**At least one parent with allergy or asthma**	222 (57.8)	230 (59.4)	452 (58.6)
**No information**	25 (6.5)	23 (5.9)	48 (6.2)
**Ethnicity of parents**			
**Western**	116 (30.2)	112 (29.0)	228 (29.6)
**Non**-**western**	182 (47.4)	205 (53.0)	387 (50.2)
**No information**	86 (22.4)	70 (18.1)	156 (20.2)

* Range of age 1–5 in all groups.

**Cough or wheeze or shortness of breath.

*** Cough & wheeze or cough & shortness of breath or wheeze & shortness of breath.

**** Cough & wheeze & shortness of breath.

# Depending on age at onset, a participant was intended to fill in 2, 3 or 4 questionnaires.

**Table 2:** Baseline characteristics of the gift voucher trial

	**Gift Voucher**	**No Gift Voucher**	**Total**
**N**	389	382	771
**Male**, **n (%)**	230 (59.1)	202 (52.9)	432 (56)
**Age yrs**, **mean **(**SD**)*	2.22 (1.2)	2.33 (1.3)	2.27 (1.2)
**Nr of symptoms at onset**, **N (%)**			
**1** **	263 (67.6)	255 (66.8)	518 (67.2)
**2** ***	94 (24.2)	101 (26.4)	195 (25.3)
**3** ****	32 (8.2)	26 (6.8)	58 (7.5)
**Nr of Questionnaires**^#^			
**2**	13 (3.3)	25 (6.5)	38 (4.9)
**3**	38 (9.8)	46 (12.0)	84 (10.9)
**4**	338 (86.9)	311 (81.4)	649 (84.2)
**Level of education of parents**, **N (%)**			
**Lowest**	11 (2.8)	5 (1.3)	16 (2.1)
**Low**	66 (17.0)	51 (13.4)	117 (15.1)
**Medium**	149 (38.3)	141 (36.9)	290 (37.6)
**High**	141 (36.3)	156 (40.8)	297 (38.5)
**No information**	22 (5.7)	29 (7.6)	51 (6.6)
**Allergy or asthma parents**			
**At least one parent with allergy or asthma**	223 (57.3)	229 (59.9)	452 (58.6)
**No information**	21 (5.4)	27 (7.1)	48 (6.2)
**Ethnicity of parents**			
**Western**	120 (30.9)	108 (28.3)	228 (29.6)
**Non**-**western**	199 (51.2)	188 (49.2)	387 (50.2)
**No information**	70 (18.0)	86 (22.5)	156 (20.2)

* Range of age 1–5 in all groups.

**Cough or wheeze or shortness of breath.

*** Cough & wheeze or cough & shortness of breath or wheeze & shortness of breath.

**** Cough & wheeze & shortness of breath.

# Depending on age at onset, a participant was intended to fill in 2, 3 or 4 questionnaires.

Table 1 shows the baseline characteristics of the comparison groups of the daytrip trial and Table 2 of the gift voucher trial.

### Results of daytrip trial

Table 3 shows that the effects of the daytrip intervention were very close to zero for all outcome measures and that the 95% confidence intervals excluded any important effects (upper confidence limit corresponding with a NNT of 2,359) [6]. An exception was the probability of withdrawal which seemed slightly higher in the intervention group than in the control group (RD 0.05; Number Needed To Treat for one to Withdraw 19).

**Table 3:** Results of the analysis

**Crude effects of intervention on outcome**			
**Intervention**	**Daytrip**		
**Outcome**	**RD**	**95% CI**	**NNT**
All questionnaires returned (100% returned)	−0.008	−0.07 – 0.06	126 (NNH*)
All questionnaires returned without reminder	0.019	−0.03 – 0.07	51
Non-response	0.0004	−0.03 – 0.03	2,359
Withdrawal	0.05	−0.01 – 0.09	19
**Outcome**	**Median**-**difference intervention group**-**control group**	**p**-**value of ranksum**-**test**	
Number of reminders sent	0.5	0.753	
Percentage of all questionnaires returned	0	0.803	
**Intervention**	**Gift voucher**		
**Outcome**	**RD**	**95% CI**	**NNT**
All questionnaires returned (100% returned)	0.017	−0.50 – 0.08	60
All questionnaires returned without reminder	0.015	−0.04 – 0.07	67
Non-response	−0.017	−0.05 – 0.02	60 (NNH*)
Withdrawal	0.008	−0.03 – 0.05	118
**Outcome**	**Median**-**difference intervention group**-**control group**	**p**-**value of ranksum**-**test**	
Number of reminders sent	1	0.045	
Percentage of all questionnaires returned	0	0.374	
**Outcome**	**Odds Ratio**	**p**-**value of multilevel logistic regression**	**95% CI**
Response Rate per questionnaire	1.29	0.26	0.83 – 2.01

Table 3 presents the results of the analysis on “all questionnaires returned, “without reminders” versus “not all questionnaires returned or reminders sent”. Risk Differences (RD) with 95% confidence intervals and Number Needed Treat (NNT = reciprocal of RD) for are presented.

CI 95%-level of NNT is not given, because all outcomes are not significant. In case of a non-significant treatment effect at 5%, the CI95% for the risk difference will include zero, and thus the 95% confidence interval for the number needed to treat will include infinity (∞). For example the effect of daytrip on non-responders gives an RD of 0.0004 (CI95% -0.03 – 0.03; including zero). The NNT is 2359 (1/0.0004), if we calculate CI95% on the same way, it must include infinity, thus from 32 to ∞ and from minus 31 to minus ∞ [6].

* Number needed to harm (NNH in case of a negative risk difference).

The median percentage of all questionnaires received was 100 (IQR 75–100) in the daytrip group and 100 (IQR 75–100) in the control group. Median number of reminders sent was 2.5 in the daytrip group and 3 in the control group. The effect of the daytrip intervention on the percentage of all returned questionnaires and the number of reminders in the groups did not differ significantly (Wilcoxon P values 0.75 and 0.80, respectively).

### Results of gift voucher trial

Table 3 shows that the effects of the voucher intervention were also very close to zero for almost all outcome measures, except for the number of reminders. Median numbers of reminders were 2 in the voucher group and 3 in the control group. Wilcoxon rank-sum test on the effect of the intervention on the number of reminders in the groups was significant (p 0.045). The median ‘percentage of all questionnaires received’ was 100 (IQR 75–100) in the voucher group and 100 (IQR 67–100) in the control group. The effect of the voucher intervention on ‘percentage of all questionnaires received’ was not significant (Wilcoxon P value 0.37).

Multilevel logistic regression analysis of the effect of the gift voucher intervention on the response rate per questionnaire round showed a 29% larger odds of response due to the voucher, but that effect was estimated imprecisely as the confidence interval shows (OR 1.29; 95% CI 0.83 – 2.01; ICC 0.613).

### Costs of intervention

In the daytrip trial we eventually awarded the daytrip 4 times, including entrance tickets and transportation, with a total cost of €645. In the gift voucher trial, in total, we raffled 40 vouchers, worth €12.50, a total amount of €500.

## Discussion

### Main findings

We found that two different lottery-style non-monetary incentives did not improve response rates on postal questionnaires in a longitudinal cohort study. We hypothesized that a lottery-style incentive could positively affect the different endpoints and response rates in particular. Why might these interventions not have worked? We offer the following interpretations for the daytrip trial and the voucher trial respectively. As for the daytrip trial, first, the daytrip could only be won if all questionnaires were returned completely. Once one deadline was missed the chance to win was zero and this realization might have taken away further motivation.

Due to the factorial design, participants in one intervention group (VD) had the chance to win both the voucher(s) and a daytrip. For the main outcome, we assessed the presence of a synergistic effect between the two lotteries and, in line with the literature, estimated the regression coefficient of a dummy variable representing the interaction using logistic regression [7]. We expected to find either no interaction between the two lotteries or a synergistic effect. The latter may have been present since those eager to win vouchers would automatically be candidates for a daytrip and vice versa. However, we found evidence of an antagonistic effect (OR 0.55, 95% CI 0.30-0.99, p = 0.048). The implication is that the effect of the two lotteries combined is less than that of the product of the two separate effects (multiplicative scale due to the logistic regression model). We could not think of a plausible mechanism for this to happen and therefore – in a Bayesian way of thinking – we decided that given the small prior likelihood of an antagonistic effect combined with moderately weak evidence (p = 0.048) of antagonism, the posterior likelihood of the hypothesis that antagonism is present was small. Therefore we decided to analyse the two interventions separately [7-10]. It is important to realize that even if the daytrip effect might have been reduced as follow-up became longer – after at least one deadline had been missed – this does not imply a differential effect across the groups and cannot explain a negative interaction.

### Strengths and limitations

Our study has several strengths. First, all participants were unaware of participating in the trial(s). This prevented information bias. Second, because the number of children was relatively large, the randomization resulted in comparable groups in both trials. In particular, the follow-up times were comparable across groups and confounding by different numbers of questionnaires is unlikely. Third, the daytrip incentive in particular was suitable to the participants who all had at least one young child and possibly more. Fourth, for the staff it was relative easy to add a contest to the questionnaires and the extra costs were acceptable and may be easy to afford in other studies. Fifth, almost all RCTs on methods for increasing response rates in postal questionnaires are performed in cross-sectional settings or studies with a short follow-up. Our design makes these results more generalizable for other longitudinal cohort studies. Sixth, we checked interaction between the two interventions in line with advice from the literature and we examined several relevant intervention-covariable interactions. For example, it is imaginable that participating families with lower level of education or non-western ethnicity earn less and therefore be more susceptible to these interventions than highly educated or western participants [11,12].

We see the following limitations to the study. First, participants of ARCADE come from primary care and are generally not severely ill and therefore possibly less concerned with the study and its results. Thereby, participants are young and mainly living in busy families with young children, making participation in this type of research perhaps not the highest priority or at least challenging. Second, research staff was not blinded for the randomization during the follow up period, although this information was kept in another worksheet in the database than necessary for daily use and information about the randomization was not in their interest.

Third, participants did not know how large the chances of winning were and this uncertainty may have had a demotivating effect. Unconditional fixed payments could be more effective [13]. Finally, we had planned time-to-event analyses to assess the intervention effects on return times of questionnaires and on the degree of completeness of questionnaires. However, due to suboptimal logistical and data-entry procedures, we were unable to perform these analyses. However, given the negative results on all endpoints, we do not expect that the ones omitted would have shown a different picture.

### What others found and how this fits in

In a meta-analysis of Booker et al. on monetary or non-monetary incentives used in postal questionnaires in longitudinal research, increases of the response and retention rates, if monetary incentives were received, were found. The authors claim an increase of the odds by more than half [1]. We found that these positive effects of monetary incentives may not generalize to non-monetary incentives. Booker et al. were inconclusive with regard to cash versus gift incentives.

### Implications for further research

There is a lack of evaluations of (non-)monetary incentive strategies to increase response rates or retention rates in longitudinal research. Increasing response rates possibly has a beneficial effect on job satisfaction for the research group, due to decreasing workload as a consequence of non-responders (telephone calls, reminders, etc.). In addition, narrower confidence intervals due to larger numbers and better cost effectiveness may be obtained. Our interventions, in lottery-style did not lead to the hypothesized beneficial effects, but the method of a nested RCT in our cohort study may encourage others in longitudinal research to test different strategies to increase response rates. For instance, it would be interesting to evaluate non-lottery-style interventions, for example a gift voucher for everyone who returns the questionnaire.

## Conclusion

Our findings do not support the idea that monetary lottery-style incentives reduce loss to follow-up, the need for reminders and to increase response rates in observational cohort studies with follow-up durations of around 2 years.

## Competing interests

There were no competing interests.

## Authors’ contributions

LvdM had the lead, participated in the design of the study and protocol, carried out the protocol, performed analysis and drafted the manuscript. KvW participated in the design of the study, had the lead of the cohort during the trial period and revised the manuscript critically. JM participated in the design of the study, performed randomization, managed the dataset during the data collection period and revised the manuscript critically. PB participated in the design of the study and revised the manuscript critically. MP participated in the protocol of the methodology of the study and revised the manuscript critically. GtR conceived the study, participated in the design of the study and coordination, participated in the statistical analysis and helped to draft the manuscript. All authors read and approved the manuscript.

## Pre-publication history

The pre-publication history for this paper can be accessed here:

http://www.biomedcentral.com/1471-2288/12/186/prepub
